# Ease of Intubation with McGrath Videolaryngoscope and Incidence of Adverse Events During Tracheal Intubation in COVID-19 Patients: A Prospective Observational Study

**DOI:** 10.2478/jccm-2023-0020

**Published:** 2023-07-31

**Authors:** Faisal Shamim, Muhammad Sohaib, Khalid Samad, Muhammad Faisal Khan, Adil A. Manji, Asad Latif

**Affiliations:** Aga Khan University Hospital, Karachi, Pakistan

**Keywords:** McGrath, videolaryngoscope, tracheal intubation, COVID-19, first-pass success, Percentage of Glottic Opening (POGO)

## Abstract

**Background:**

Tracheal intubation in critically ill patients remains high-risk despite advances in equipment, technique, and clinical guidelines. Many patients with COVID-19 were in respiratory distress and required intubation that is considered an aerosol-generating procedure (AGP). The transition to videolaryngoscopy as a routine first line option throughout anesthetic and ICU practice has been reported. We evaluated the ease of intubation, success rate, use of accessory maneuvers and adverse outcomes during and 24 hours after intubation with the McGrath videolaryngoscope.

**Methods:**

This was a prospective, observational single center study conducted at non-operating room locations that included all adults (>18 years old) with suspected or confirmed COVID-19 infection and were intubated by McGrath videolaryngoscope. The anesthesiologist performed tracheal intubation were requested to fill online data collection form. A co-investigator was responsible to coordinate daily with assigned consultants for COVID intubation and follow up of patients at 24 hours after intubation.

**Results:**

A total of 105 patients were included in our study. Patients were predominantly male (n=78; 74.3%), their COVID status was either confirmed (n=97, 92.4%) or suspected (n=8, 7.6%). Most were intubated in the COVID ward (n=59, 56.2%) or COVID ICU (n=23, 21.9%). The overall success rate of intubation with McGrath in the first attempt was 82.9%. The glottic view was either full (n=85, 80.95%), partial (n=16, 15.24%) or none (n=4, 3.81%). During intubation, hypoxemia occurred in 18.1% and hypotension in 16.2% patients. Within 24 hours of intubation, pneumothorax occurred in 1.9%, cardiac arrest and return of spontaneous circulation in 6.7% and mortality in 13.3% of patients.

**Conclusion:**

These results illustrate the ease and utility of the McGrath videolaryngoscope for tracheal intubation in COVID-19 patients. Its disposable blade is of significant value in protectin during tracheal intubation.

## Introduction

The novel coronavirus COVID-19 has caused a pandemic of previously unimaginable proportions. Highly infective, it is leading to an extremely high absolute burden of morbidity and mortality, primarily due to hypoxic respiratory failure, hemodynamic instability, reduced oxygen stores and increased oxygen consumption [1]. Many patients with COVID-19 were in critical condition and require intubation, an aerosol-generating procedure with a high propensity to generate enormous viral loads [2]. Moreover, increased risk of aerosol transmission during airway management is also possible due to the proximity of the intubator to the patient's airway [2,3]. During the SARS outbreak in 2003, healthcare workers who performed aerosol-generating procedures such as tracheal intubation had a higher likelihood of contracting the disease, compared to non-intubator [4]. Therefore, certain attending healthcare providers like anesthesiologist, intensivists, and emergency physicians are particularly at higher risk of nosocomial infection not only due to the aerosolization of the virus during tracheal intubation but also due to increased duration of exposure [5,6].

Tracheal intubation in critically ill patients remains high-risk despite advances in equipment, technique, and clinical guidelines. Peri-intubation cardiac arrest occurs in about 1% of cases and is highly associated with peri-intubation oxygen desaturation and hypotension [7]. Moreover, hypoxemia and hemodynamic instability before intubation are associated with increased risk of complications. Videolaryngoscopy therefore becomes an important tool in anticipated and unanticipated difficult intubation. Videolaryngoscopes (VL) are designed to improve visualization of the glottis, increase first attempt intubation success rate, reduce intubation related complications, and minimize exposure by increasing the distance between the operator and the patient's oral cavity [8]. The transition to videolaryngoscopy as a routine first line option through anesthetic and ICU practice has been reported and is currently suggested by many societies as a primary intubation device for COVID patients [9]. One such videolaryngoscope is the McGrath MAC (Aircraft Medical, Edinburgh, Scotland), a unique battery-powered device with attached screen and disposable laryngoscope blade that actually have made its worldwide importance in current pandemic.

We aimed to assess ease of intubation and first pass success with McGrath videolaryngoscope in COVID-19 patients. Secondary objectives were use of accessory maneuvers and incidence of major adverse events within 24 hours of intubation.

## Materials and Methods

This prospective observational study was conducted from October 2020 to January 2022 after approval by institutional ethics review committee (ERC). We included all consecutive adults (>18 years old) suspected or confirmed COVID-19 patients undergoing emergent or urgent tracheal intubation by McGrath videolaryngoscope and excluded any patients undergoing intubation for general anesthesia. Study locations included the Intensive Care Unit (ICU), Acute Care Unit (ACU), Emergency Room (ER) and COVID ward at the Aga Khan University Hospital, Karachi. Waiver of patient consent was obtained from ERC. Each patient was coded through a computer-generated patient identification number (PIN) and no identifiers like patient name, initials or hospital record numbers were used. Data was collected via an online form and anesthesiologists performed tracheal intubation were requested to fill. We identified data coordinators among investigators who contacted the consultants on COVID intubation calls. The data coordinators were responsible for data collection regarding complications and adverse events by follow up of patients at 24 hours after intubation through bedside visit and chart review.

The primary outcome was to determine ease of intubation by first pass success using McGrath VL in COVID-19 patients outside operating room. Secondary outcomes included best glottic view by percentage of glottic score (POGO), use of accessory maneuvers, adjuncts used, number of intubation attempt and major adverse events during and within 24 hours of intubation [10]. Data was collected through anonymized online form. This included patient characteristics (Age [more than or less than 65 years], gender), comorbidities, patients COVID status, ongoing oxygen therapy before intubation, patient position for intubation, induction technique (classic or modified rapid sequence induction), rescue devices or techniques, and time taken to successfully pass endotracheal tube. Classic RSI mainly consists of preoxygenation, administration of short acting anesthetic/sedative agents, rapidly acting muscle relaxant, application of cricoid pressure, laryngoscopy and inserting a cuffed endotracheal tube to secure the airway. Modified rapid sequence induction (RSI) is implemented in an attempt to optimize patient outcomes and reduce excess risk exposure [11]. In addition to above components of classic RSI, the modification may include use of short acting opioids, ketamine or lidocaine, use of positive pressure ventilation before passing tracheal tube or apneic oxygenation. Glottic view taken according to modified POGO score as full (100%), partial (50%) and nil (0%). Ease of intubation was described as easy (ETT passed in first attempt), Modified (ETT passed with more than one attempt, or a modified technique or adjunct used) and unachievable (unable to pass ETT). Accessory maneuver was defined as external laryngeal manipulation or use of Magill forceps, or both required to facilitate passage of endotracheal tube through glottic opening. Hypoxemia defined as oxygen saturation (SaO2) <90%. Hypotension was described as having a systolic blood pressure of less than 90 mmHg or mean arterial pressure (MAP) of less than 60 mmHg.

All analysis was performed using statistical packages for social science version 19 (SPSS Inc., Chicago, IL). Clinical features of patients were characterized using descriptive statistics and comparisons of categorical variables was performed using chi-square or Fisher's exact test at a two-sided significance level of 0.05.

## Results

### Baseline characteristics

In our cohort of 105 patients, 74.3% were male. There were 45 (42.9%) patients who were more than sixty-five years and 60 (57.1%) were less than 65 years. Consultant anesthesiologist performed all intubations. Patient's COVID status was either identified as confirmed (n=97, 92.4%) or suspected (n=8, 7.6%). Classic rapid sequence induction (RSI) was the most employed technique in 71 (67.6%) patients while few consultants did modified RSI in 21 (20%) patients. Facemask holding technique during preoxygenation, or anesthesia induction was by single hand (n=66, 62.9%), both hands CE (n=36, 34.3%) or both hands VE (n=3, 2.9%). The baseline characteristics and induction technique are detailed in Table 1. Fisher exact test was used for evaluating an association between “Ease of Intubation” and “Gender”, “Age”, “Glottis View”, “Face Mask Holding” and “Patient Position” at a two-sided significance level of 0.05. The test was significant for an association between “Ease of Intubation” and “Age,” “Patient Position” and “Glottis View” with P-values of 0.018, 0.002 and 0.001, respectively indicating that there is an association between “Ease of Intubation” and the “Age”, “Patient Position” and “Glottis View”.

**Table 1.** Demographics and Baseline characteristics

**Variables**	**Categories**	**n**	**(%)**
**Gender**	Male	78	74.3
	Female	27	25.7

**Age groups (years)**	Less than 65	60	57.1
	More than 65	45	42.9

**Comorbidities**	Diabetes mellitus	70	66.7
	Hypertension	56	53.3
	Ischemic heart disease	23	21.9
	Immune compromise status	9	8.6
	CKD	9	8.6
	None	6	5.7
	COPD	3	2.9
	Malignancy	2	1.9

**Patient's COVID status**	Confirmed	97	92.4
	Suspected	8	7.6

**Location**	COVID ward	59	56.2
	COVID ICU	23	21.9
	Acute care unit	11	10.5
	Emergency room	6	5.7
	Others	4	3.8
	Surgical or medical ICU	2	1.9
	COVID operating room	2	1.9

**Ongoing oxygen therapy before intubation**	Conventional nasal cannula / prongs	4	3.8
	Simple facemask / non-rebreather mask	15	14.3
	High flow nasal oxygen / cannula	0	0
	Continuous positive airway pressure	9	8.6
	Non-invasive positive pressure ventilation	77	73.3

**Patient position for intubation**	Supine	74	70.5
	Head up 15–30 degree	29	27.6
	RAMP	2	1.9

**Induction technique**	Classic RSI	71	67.6
	Modified RSI	21	20.0
	Cardiac arrest situation - no drugs given	13	2.4

### Induction and Intubation

The glottic view with McGrath VL as per POGO score was full in 80.95% patients. 16 cases had partial (15.24%) and none was seen in 4 patients (3.81%). Successful intubation with first attempt was noted in 87 cases. Intubation was reported easy (92.4%) in full and partial glottic view (n=84, 98.8% and n=13, 81.3% respectively). Table 2 shows data regarding ease of VL insertion and successful intubation. Regardless of glottic view, none of the consultants reported impossible to intubate a patient utilizing McGrath videolaryngoscope.

**Table 2.** Tracheal intubation data

**Variables**	**n**	**(%)**
**Glottic View**		
Full	85	8
Partial	16	15.2
None	4	3.8

**Ease of intubation**		
Easy – ETT passed in first attempt	87	82.9
Modified – ETT passed with more than one attempt	18	17
Unachievable – unable to pass ETT	0	0

**Accessory maneuvers/adjuncts**		
External laryngeal manipulation	23	21.9
ETT stylet	68	64.8
Bougie	5	4.8
Magill forceps	0	0
None	24	22.9

**Rescue device(s) and techniques**		
Change to conventional laryngoscope	7	6.7
LMA	6	5.7
Face mask ventilation	5	4.8
Front of neck access	0	0
None	91	86.7

**Successful intubation**		
First attempt	87	82.9
Second attempt	14	13.3
More than two attempts	4	3.8

Adjuncts required to facilitate intubation during McGrath videolaryngoscopy were ETT stylet that was used in 68 (64.7%) patients. The high number of stylets use is according to VL manufacturer recommendation to aid intubation. Five (4.76%) cases required bougie especially in three patients where glottic view is none. External laryngeal manipulation needed in 14 (16.5%) patients with full glottic view and 6 (37.5%) with partial glottic view. Twenty-four (22.9%) patients did not require any accessory maneuver or adjunct.

McGrath videolaryngoscope allowed successful intubation in the first attempt in a cumulative of 97 patients (92.3%) – [83 (97.64%) patients with full glottic view, 13 (81.3%) patients with partial glottic view and 1 (25%) patient with no glottic view]. Only 3 patients (2.86%) required more than two attempts for successful intubation, of which 1 patient had partial glottic view (6.3%) and two had no glottic view (50%). Out of 18 patients who required second or more than two attempts, rescue device or techniques employed to make a successful intubation. On seven occasions (6.7%), conventional laryngoscope was chosen while supraglottic airway used in six (5.7%) cases (Table 3).

**Table 3.** Association of ease of intubation based on Fishers exact test

**Variables**	**Ease of Intubation**			

	**Easy**	**Modified**	**Total**	**P-value**
**Gender**				
Male	66 (84.6%)	12 (15.4%)	78	0.554
Female	21 (77.8%)	6 (22.2%)	27	

**Age**				
Less than 65	45 (75%)	15 (25%)	60	0.018
More than 65	42 (93.3%)	3 (7.6%)	45	

**Patient Position**				
Supine	57 (77%)	17 (23%)	74	0.002
Head up	29 (100%)	0	29	
RAMP	1 (50%)	1 (50%)	2	

**Face Mask Holding**				
Single Hand	53 (80.3%)	13 (19.3%)	66	0.774
Both Hands CE	31 (86%)	5 (14%)	36	
Both Hands VE	3 (100%)	0	3	

**Glottis view**				
Full	75 (88.2%)	10 (17.8%)	85	0.001
Partial	12 (75%)	4 (25%)	16	
None	0	4 (100%)	4	

### Adverse events and complications

**Fig. 1.**
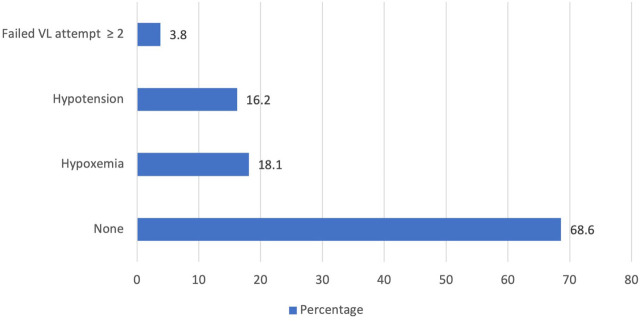
Adverse effects observed during intubation

**Fig. 2.**
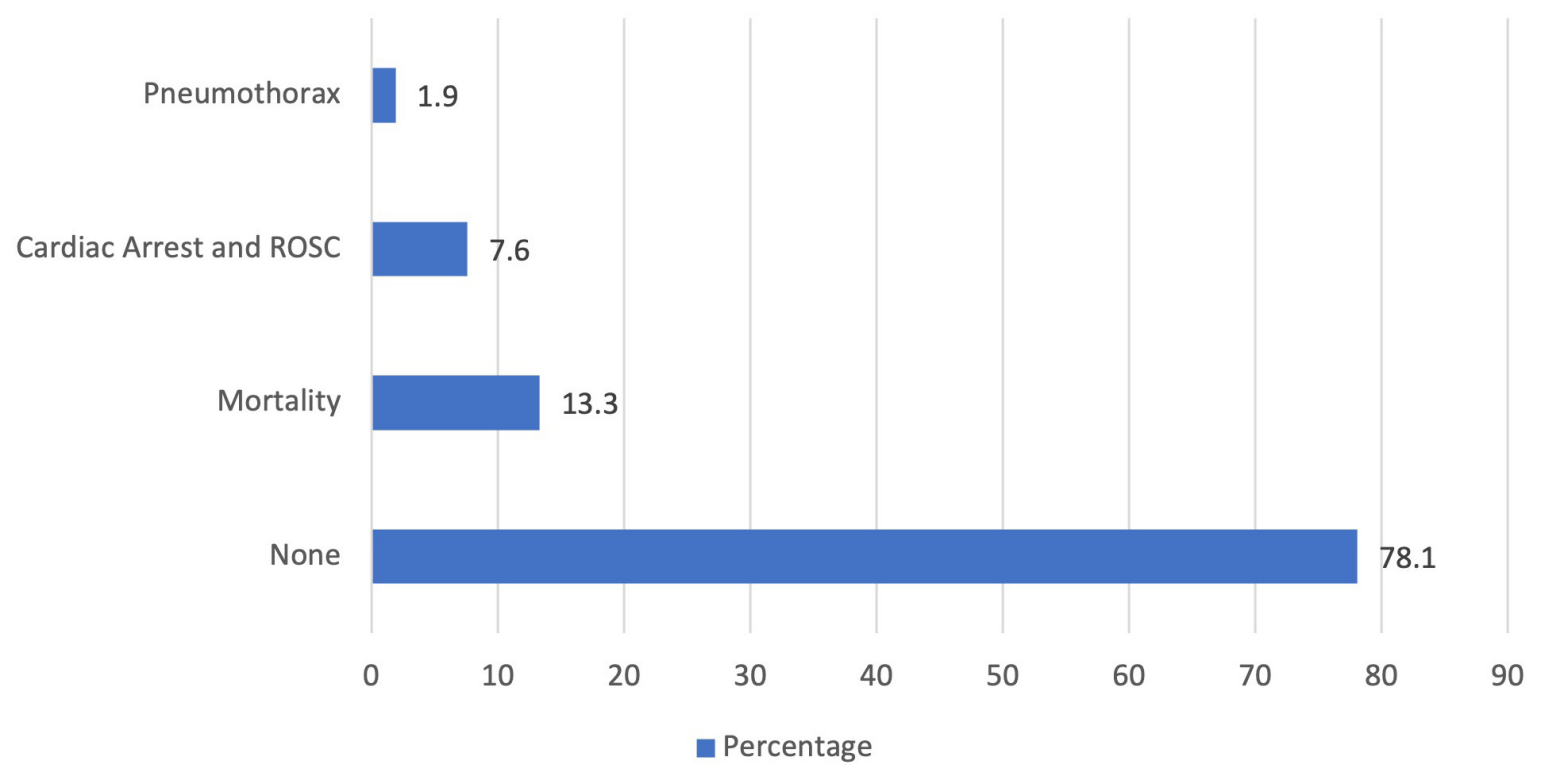
Adverse effects observed within 24 hours of intubation

Adverse events and complications were categorized into those arising during intubation and within 24 hours (Figure 1). During intubation, hypoxemia occurred in 18% and hypotension in 16.2% of patients. Intubation required two or more attempts in 2.9% of patients. 69.5% of patients were intubated successfully in the first or second attempt without any hypoxemia or hypotension. Within 24 hours of intubation (Figure 2), pneumothorax occurred in 1.9% of patients, cardiac arrest and return to spontaneous circulation in 7.6% of patients, and mortality in 13.3% of patients. None of these three complications were reported in 78.1% of patients within 24 hours of intubation.

## Discussion

Patients with suspected or confirmed COVID-19 are at greater risk for complicated intubation because not only is it a common occurrence that these patients decline rapidly due to a lack of inspiratory reserve, but strict hospital COVID infection control policies often make it difficult if not impossible to perform adequate airway evaluation or have the necessary number of supportive personnel present to assist when urgent intubation is required. Outside the operating room, tracheal intubation is commonly performed by personnel with less experience in airway management, compared to anesthesiologists, and this can make intubation more arduous of a task, potentially even compromising patient safety.

Videolaryngoscopy is becoming increasingly popular for use in both normal and difficult airways, especially since the beginning of the pandemic. The difficult airway algorithm developed by the American Society of Anesthesiologists (ASA) does not endorse nor discourage the use of videolaryngoscopy and instead leaves it to the anesthesiologist to decide if and when to use it [13]. Moreover, the Difficult Airway Society guidelines state that videolaryngoscopy use is dependent upon good clinical judgement, and that all anesthesiologists should have immediate access to videolaryngoscopes [14]. However, especially in an anticipated difficult airway, the advantages and practicality of videolaryngoscopy makes it an excellent tool for intubation, with some authors even recommending it for first line use in both normal as well as difficult airways [15]. Our data will be useful for future planning and management of such patients and help establish precautions for staff. The recommendations are urgently needed and will suggest good clinical practice for our local healthcare facilities

Our study exemplifies an overall high success rate of intubation with McGrath videolaryngoscope with only 2.9% of patients requiring more than two attempts to intubate successfully, which is comparable to the results of other studies. These studies reported 100% success with direct laryngoscopy in simulated difficult airway patients [16], 98–100% in patients with normal airway [17], and 95% of patients after failed direct intubation [18]. These high success rates are likely due to videolaryngoscopy facilitating the assistant as well at the time of intubation by allowing better shared visualization of the glottis, since both the screen and the blade can be viewed during insertion, without needing to align the oral, pharyngeal, or laryngeal axes. Moreover, it allows better coordination between the anesthesiologist and the assistant, and correction of laryngoscopic maneuvers if required.

Despite an overall success rate, accessory adjuncts and maneuvers were required to facilitate difficult intubation in 77.1% of patients, most of whom had full (80.95%) or partial (15.2%) glottic views, as opposed to 3.8% of patients with no glottic view, none of which were easy intubations. Three patients, all intubated via classic RSI, required more than two attempts with videolaryngoscope for intubation, as well as accessory maneuvers. The first was a female of less than 65 years with no known comorbid conditions, confirmed COVID, no glottic view and had required external laryngeal manipulation, ETT stylet and Bougie, as well as a change to conventional laryngoscope for successful intubation which took thirty seconds to one minute. There were no post intubation complications after 24 hours. Another immunocompromised male of less than 65 years with Diabetes Mellitus, no glottic view and confirmed COVID required External laryngeal manipulation, Bougie and face mask ventilation before successful intubation which took more than 2 minutes and had developed hypoxemia during intubation. Again, there were no post intubation complications after 24 hours. Lastly, one female of less than 65 years with chronic kidney disease, confirmed COVID, partial glottic view and had required External laryngeal manipulation and ETT stylet for successful intubation, which took one to two minutes, and had developed hypoxemia during intubation. 24-hour post intubation revealed mortality. Other studies have reported a 24h mortality of 10.4% [19]. These findings may be related to patient characteristics or events at tracheal intubation, but further analysis of this cannot be performed due to having only observational data.

More than half of all patients were intubated within 30 seconds and rest under a minute, portraying that the majority were intubated quickly. However, other studies show that use of videolaryngoscopy results in either no difference in time to intubation [20] or longer intubation time compared to traditional laryngoscopes [16,20].

Anesthesiologists in our study reported easy McGrath-assisted intubation in 92.4% of cases and were able to successfully intubate using the McGrath in all but seven patients, demonstrating a considerable advantage in difficult cases. One randomized control trial comparing six different videolaryngoscopes found the McGrath to have the highest first attempt intubation success rate [21,22].

### Limitations

As our study did not contain patients with airway injury or patients from the pediatric population, and the fact that our study was performed at a single center, our findings cannot be generalized to these patient subgroups. Our results may also be limited due to the experience of the intubator, as the anesthesiologists at our facility have had regular practice managing normal and difficult airways in both high and low risk patients and could not be blinded for this study. As our study also uses a questionnaire with some subjective variables, recall bias and self-reporting may have occurred.

## Conclusion

The findings of our research demonstrate the effectiveness, feasibility and high rate of success using McGrath videolaryngoscope to facilitate tracheal intubation in suspected or confirmed COVID-19 patients. Videolaryngoscopy improve first attempt success in difficult airways and is recommended when available. We would suggest that McGrath VL with a disposable blade design is better suited for intubation in patients during the pandemic with highly infectious diseases.

## References

[1] Centers for Disease Control and Prevention Cases and latest updates.

[2] Meng L, Qiu H, Wan L (2020). Intubation and Ventilation amid the COVID-19 Outbreak. Anesthesiology..

[3] Wax RS, Christian MD (2020). Practical recommendations for critical care and anesthesiology teams caring for novel coronavirus (2019-nCoV) patients. Can J Anesth..

[4] Tran K, Cimon K, Severn M, Pessoa-Silva CL, Conly J (2012). Aerosol Generating Procedures and Risk of Transmission of Acute Respiratory Infections to Healthcare Workers: A Systematic Review. PLoS One..

[5] Chen X, Liu Y, Gong Y (2020). Perioperative Management of Patients Infected with the Novel Coronavirus: Recommendation from the Joint Task Force of the Chinese Society of Anesthesiology and the Chinese Association of Anesthesiologists. Anesthesiology..

[6] Odor PM, Neun M, Bampoe S (2020). Anaesthesia and COVID-19: infection control. Br J Anaesth..

[7] April MD, Arana A, Reynolds JC (2021). Peri-intubation cardiac arrest in the Emergency Department: A National Emergency Airway Registry (NEAR) study. Resuscitation..

[8] Chemsian R, Bhananker S, Ramaiah R (2014). Videolaryngoscopy. Int J Crit Illn Inj Sci..

[9] Cook TM, El-Boghdadly K, McGuire B, McNarry AF, Patel A, Higgs A (2020). Consensus guidelines for managing the airway in patients with COVID-19: Guidelines from the Difficult Airway Society, the Association of Anaesthetists the Intensive Care Society, the Faculty of Intensive Care Medicine and the Royal College of Anaesthetists. Anaesthesia..

[10] Levitan RM, Ochroch EA, Kush S, Shofer FS, Hollander JE (1998). Assessment of airway visualization: validation of the percentage of glottic opening (POGO) scale. Acad Emerg Med..

[11] Lyon RM, Perkins ZB, Chatterjee D (2015). Significant modification of traditional rapid sequence induction improves safety and effectiveness of pre-hospital trauma anaesthesia. Crit Care..

[12] Yao W, Wang T, Jiang B (2020). Emergency tracheal intubation in 202 patients with COVID-19 in Wuhan, China: lessons learnt and international expert recommendations. Br J Anaesth..

[13] Apfelbaum JL, Hagberg CA, Connis RT (2022). American Society of Anesthesiologists Practice Guidelines for Management of the Difficult Airway. Anesthesiology..

[14] Frerk C, Mitchell VS, McNarry AF (2015). Difficult Airway Society 2015 guidelines for management of unanticipated difficult intubation in adults. Br J Anaesth..

[15] Cook TM, Kelly FE (2016). The Difficult Airway Society 2015 guidelines and the sacred cows of routine airway management. Anaesthesia..

[16] Taylor AM, Peck M, Launcelott S (2012). The McGrath^®^ Series 5 videolaryngoscope vs the Macintosh laryngoscope: a randomised, controlled trial in patients with a simulated difficult airway. Anaesthesia..

[17] Noppens RR, Möbus S, Heid F, Schmidtmann I, Werner C, Piepho T (2010). Evaluation of the McGrath® Series 5 videolaryngoscope after failed direct laryngoscopy. Anaesthesia..

[18] Wallace CD, Foulds LT, McLeod GA, Younger RA, McGuire BE (2015). A comparison of the ease of tracheal intubation using a McGrath MAC^®^ laryngoscope and a standard Macintosh laryngoscope. Anaesthesia.

[19] Niforopoulou P, Pantazopoulos I, Demestiha T, Koudouna E, Xanthos T (2010). Video-laryngoscopes in the adult airway management: a topical review of the literature. Acta Anaesthesiol Scand..

[20] Liu ZJ, Yi J, Guo WJ, Ma C, Huang YG (2016). Comparison of McGrath Series 3 and Macintosh Laryngoscopes for Tracheal Intubation in Patients With Normal Airway by Inexperienced Anesthetists: A Randomized Study. Medicine (Baltimore)..

[21] Walker L, Brampton W, Halai M (2009). Randomized controlled trial of intubation with the McGrath® Series 5 videolaryngoscope by inexperienced anaesthetists. Br J Anaesth..

[22] Kleine-Brueggeney M, Greif R, Schoettker P, Savoldelli GL, Nabecker S, Theiler LG (2016). Evaluation of six videolaryngoscopes in 720 patients with a simulated difficult airway: a multicentre randomized controlled trial. Br J Anaesth..

